# Study on the Product Characteristics of Pyrolysis Lignin with Calcium Salt Additives

**DOI:** 10.3390/ma12101609

**Published:** 2019-05-16

**Authors:** Yong Cui, Wenliang Wang, Jianmin Chang

**Affiliations:** 1 Precision Manufacturing Engineering Department, Suzhou Vocational Institute of Industrial Technology, Suzhou 215104, China; 2 College of Bioresources Chemical and Materials Engineering, Shaanxi University of Science & Technology, Xi’an 710021, China; wangwenliang@sust.edu.cn; 3 College of Materials Science and Technology, Beijing Forestry University, Beijing 100083, China; cjianmin@bjfu.edu.cn

**Keywords:** lignin, catalytic pyrolysis, bio-oil, calcium salt, product characteristics

## Abstract

This study investigated and compared the product characteristics of pyrolysis lignin under different catalytic effects resulting from various calcium salts. The pyrolysis of lignin was conducted in a fixed-bed reactor with calcium salt additives, which included CaCl_2_, Ca(OH)_2_, and Ca(HCOO)_2_. The compositions of gas and bio-oil were detected using gas chromatography/mass spectrometry (GC/MS). The characterizations of chars were examined using Brunauer–Emmett–Teller (BET) surface area and scanning electron microscopy (SEM). The results indicate that all three types of calcium salts helped to promote bio-oil yield and inhibit gas and char from forming. Regarding the composition of gas products, calcium salt additives increased the concentrations of H_2_ and CH_4_ while decreasing the concentration of CO. In addition, calcium salt additives facilitated the formation of phenol and alkyl-phenols in bio-oil, but reduced the yields of guaiacol and vanillin, in the order CaCl_2_ < Ca(OH)_2_ < Ca(HCOO)_2_. Furthermore, when compared with the addition of CaCl_2_, the chars prepared by the addition of Ca(OH)_2_ and Ca(HCOO)_2_ had relatively higher BET surface areas. In conclusion, Ca(HCOO)_2_ had the greatest positive influence in regard to the product quality of lignin pyrolysis whilst also elevating the yield of value-added chemicals in bio-oils.

## 1. Introduction

As the second most abundant component of lignocellulosic biomass, lignin is composed of hydroxyl phenylpropane units with 0–2 methoxyl group(s) which are linked through C–C and ether bonds. Because lignin is the only renewable aromatic resource found in nature, the conversion of lignin to value-added chemicals is a very good method for alleviating the worldwide energy crisis. However, millions of tons of lignin are abandoned annually as industrial waste (especially in the forms of black liquor from pulp and paper mill) [1,2], which not only causes serious environmental pollution, but also results in huge bioresource waste. Therefore, it is important to develop renewable and alternative technologies for the effective use of lignin resources.

Regarding this, much attention has been paid to the thermal processes (mainly pyrolysis) involved in the production of fuels and aromatic chemicals from lignin, such as phenols [3,4], aromatic hydrocarbons [5,6], and vanillin. Typically, catalytic pyrolysis was popularly regarded as a promising technical route to produce high-quality oil and gas [7,8,9,10,11,12]. Recently, many researchers have focused on lignin pyrolysis with calcium salt additives, which improves the quality of the resulting bio-oil. Zhou et al. (2015) [13] developed a simple pretreatment of lignin with Ca(OH)_2_ that made possible the continuous pyrolysis of lignin, thus producing a phenolic-rich bio-oil. They found that pretreatment with Ca(OH)_2_ reduced the melting and agglomeration behavior of lignin and promoted its depolymerization to phenolic monomers and dimers. Mukkamala et al. (2012) [14] showed that adding Ca(HCOO)_2_ to lignin before fast pyrolysis resulted in the deoxyhydrogenation of the lignin during pyrolysis. Wang et al. (2015) [15] investigated the effect of adding CaCl_2_ on the physicochemical properties of products from lignin pyrolysis and proved that the phenol content in bio-oil was significantly increased.

On the basis of these previous studies, it was revealed that adding calcium salt to lignin is an effective method for improving the quality of pyrolysis products. However, the influences of calcium salt on the physicochemical properties of pyrolysis products have so far not been fully clarified. Furthermore, the catalytic effects of different calcium salts have not been comprehensively compared. Consequently, the object of this article was to understand the catalytic effects of CaCl_2_, Ca(OH)_2_, and Ca(HCOO)_2_ on the physicochemical properties of gas, bio-oil, and char from lignin pyrolysis. In addition, the research results were expected to act as a fundamental study, demonstrating what value-added chemicals (mainly phenolic compounds) can potentially be obtained from lignin pyrolysis with these calcium salt additives. A fixed-bed reactor was used in this study, which has the advantages of a simple structure and flexible operation, compared with other pyrolysis reactors (fluidized beds, screw kilns, spouted beds, etc.). Gas chromatography/mass spectrometry (GC/MS), Brunauer–Emmett–Teller (BET), and scanning electron microscopy (SEM) methods were adopted to investigate the product characteristics of pyrolysis lignin with calcium salt additive.

## 2. Material and Methods

### 2.1. Material Preparation

The lignin was purchased from Tokyo Chemical Industry Co., Ltd (Tokyo, Japan), which was extracted from larch through the kraft process. According to Chinese Standards GB/T 17664-1999, the proximate analysis of the air-dried lignin was 66.43 wt.% volatile and moisture, 6.21 wt.% ash, and 27.36 wt.% fixed carbon. The elemental composition of the air-dried alkali lignin was 62.4 wt.% carbon, 6.14 wt.% hydrogen, 29.43 wt.% oxygen, 0.26 wt.% nitrogen, and 1.77 wt.% sulfur, which were determined by Elementar Vario EL elemental analyzer (Elementar, Shanghai, China).

Three kinds of calcium additives, including CaCl_2_, Ca(OH)_2_, and Ca(HCOO)_2_, were selected to investigate the effect on lignin pyrolysis. On the basis of the mass ratio of 1:20 between the Ca metal atom and the lignin, it was calculated that additive amounts of CaCl_2_, Ca(OH)_2_, and Ca(HCOO)_2_ were 13.9%, 9.3% and 16.3% in the lignin, respectively. The excessive impregnation method was used to add calcium salts, which were dissolved in the deionized water. The lignin was impregnated with the additive solutions by ultrasonic immersing for 0.5 h and static immersion for 12 h. Finally, the absorbed water in the lignin was removed by vacuum drying at 105 °C for 6 h, and then the test samples were obtained.

### 2.2. Experimental Methods

**Figure 1:**
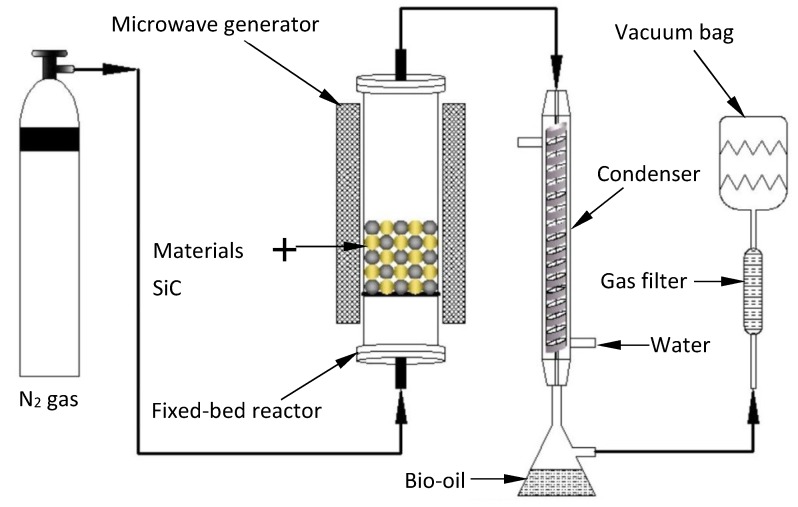
Schematic diagram of pyrolysis reactor.

Pyrolysis experiments were all carried out in the fixed-bed reactor (shown in Figure 1), which heated a certain amount of the tested sample (5 g) from ambient temperature to 600 °C at a heating rate of 20 °C/min by microwave (1000 W), where it was kept constant for 25 min [16]. Nitrogen with a flow of 1200 mL min^−1^ was used as the carrier gas. The produced pyrolysis vapors firstly passed through the condensing system with a collector to obtain liquid products (bio-oil). The uncondensed gas cleaned by the filter was stored in vacuum bags for analysis. Finally, the test samples were fully pyrolyzed and the heating temperature started to drop, but the nitrogen was still supplied to avoid oxidation until the pyrolysis residues (chars) had cooled down to room temperature. The quantity of the generated bio-oil and char was weighed, while the mass of uncondensed gas was measured by the weight difference. The water content in the bio-oil was determined with Karl Fischer Moisture Meter (ZDJ-1S, Hengteya, Taizhou, Jiangsu, China), which selected Karl reagent as the standard solution and methanol as the titrating solvent. Each test was repeated at least three times under the same conditions, and their average values were calculated and used. 

A micro-chromatograph (Agilent 4900, Santa Clara, CA, USA) was used to measure the composition of non-condensable pyrolysis gas. In order to achieve a good separation of H_2_, CH_4_, CO, and CO_2_, two specific capillary columns (Molecular sieve 5A, Plot U) were also equipped. The temperature in the inlet was kept at 100 °C while temperatures within the two columns were kept at 55 °C and 80 °C, respectively [17].

A GC/MS (Shimadzu GCMS-QP2010Plus, Shimadzu, Kyoto, Japan) was applied to identify detailed composition of the bio-oil. With a He gas flow rate of 1.0 mL min^−1^ in a split-flow ratio of 30:1, the temperature inside the inlet was kept at 280 °C. Heating temperatures were scheduled as follows: The temperatures were kept at 50 °C for 5 min, then maintained at 280 °C for 15 min as the temperature increased at a rate of 5 °C min^−1^. Junction and ion temperatures were kept at 280 °C and 250 °C, respectively, operating in an EI source electron energy of 70 eV and a scan range of 20–400 μ for mass spectrometry (MS).

The nitrogen adsorption (Quadrasorb SI, Boynton Beach, FL, USA) analyzed specific surface areas of chars obtained from lignin pyrolysis. Using the BET method, the surface area was calculated after each sample was outgassed at 77 K for 3 h. An SEM (Hitachi S-3400N, Hitachi, Tokyo, Japan) also examined the surface morphology of the chars. Before testing, all the char samples were washed with alcohol solution and later dried at 120 °C.

## 3. Results and Discussion

### 3.1. The Yields of Pyrolysis Products

**Figure 2:**
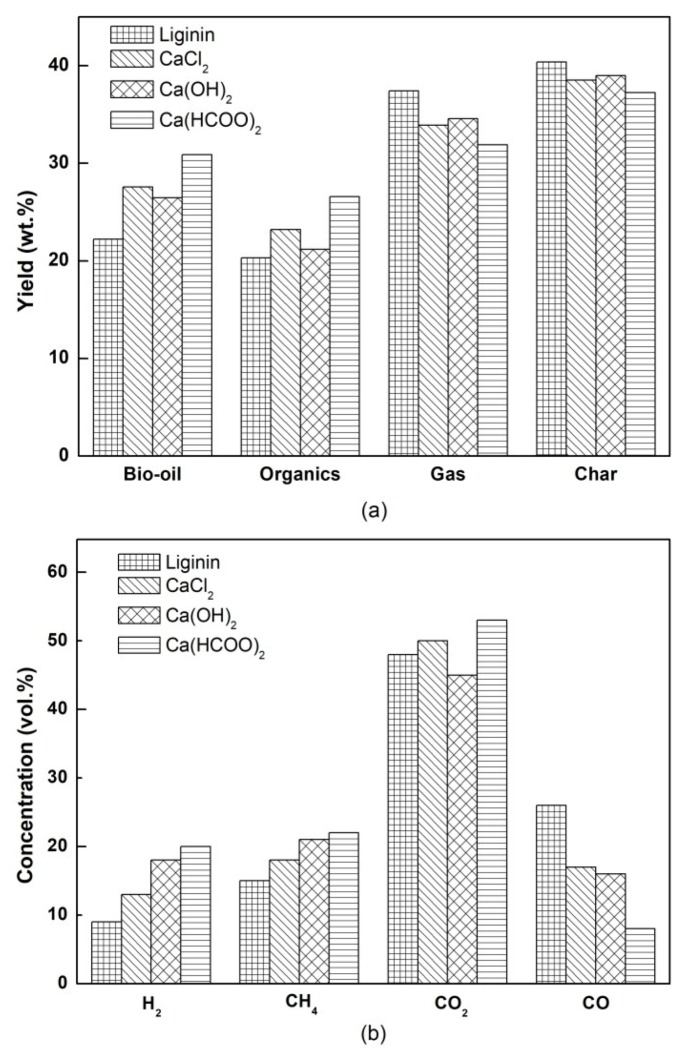
(**a**) Product yields and (**b**) gas composition in the pyrolysis of lignin with and without calcium salt additives.

As shown in Figure 2a, the pyrolysis of lignin with and without calcium additives resulted in a high char production, which was due to some very stable aromatic rings and high carbon content in the lignin [18,19]. In this study, the yields of bio-oil, gas, and char for raw lignin pyrolysis were in agreement with the results of many reports in the literature [20,21]. Figure 2a shows that the catalytic effect of three calcium salt additives led to the same tendencies in the yields of pyrolysis products, which promoted the bio-oil yields and inhibited the formation of gas and char. The influences of the three additives on product distribution were consistent with previous studies. Wang (2015) [21] proved that the catalytic pyrolysis of lignin using calcium chloride resulted in a decrease in residual carbon formation and an increase in bio-oil yield. Peng (2014) [17] reported that liquid yield increased when hydroxide alkalis was added in lignin pyrolysis, but the yields of char and gas decreased. Mukkamala (2012) [14] found that adding calcium formate to lignin during fast pyrolysis promoted the deoxyhydrogenation reaction and enhanced the liquid yield. 

Figure 2b compares the gas composition obtained from pure lignin pyrolysis and that obtained in the catalyzed pyrolysis with calcium salt. As observed, the gas fraction was mainly composed of CO_2_, with a lower contribution of CO, H_2_, and CH_4_. The results in Figure 2b revealed that all of the calcium salt additives increased the concentrations of H_2_ and CH_4_ when compared with those corresponding to the pyrolysis of pure lignin, but decreased the concentrations of CO. According to previous studies, the generation of H_2_ results from the rearrangement of the Aromatic rings to form a more organized structure [22], while the releasing of CO_2_, CO, and CH_4_ are attributable to the fragmentation of the propyl chains and the substitution of the methoxyl groups of the aromatic rings [23,24]. From the higher H_2_ and CH_4_ contents in the gas, it can be deduced that cracking of lignin into small-molecule gases was enhanced in the pyrolysis process due to the catalytic effect of the calcium salt additives. Furthermore, the lower CO content indicates that the calcium salts were active in a water–gas shift reaction, which can convert the CO into CO_2_ and H_2_ [25]. Of course, this reaction can also contribute to the higher H_2_ and CO_2_ content in the gaseous fraction. In addition, Ca(HCOO)_2_ can lead to the production of H_2_ and CO_2_ due to thermal decomposition at high pyrolysis temperatures. The reduced gas volume of CO_2_ in the case of Ca(OH)_2_ could be related to absorption by alkalis.

### 3.2. The Chemical Composition of Bio-Oil

**Figure 3:**
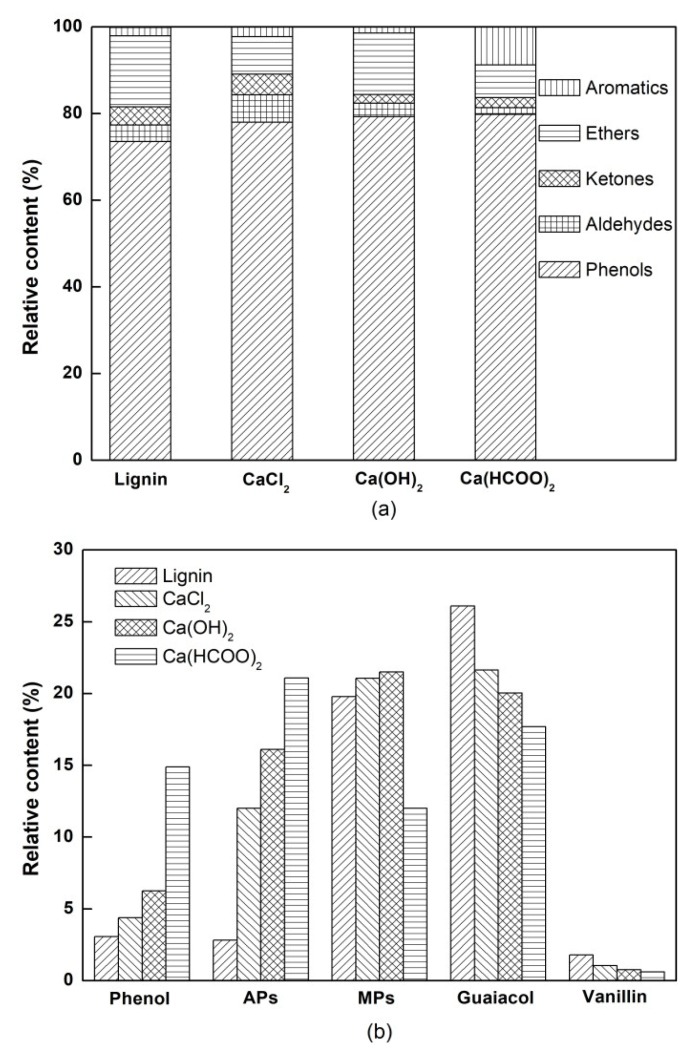
(**a**) Bio-oil composition and (**b**) contents of major phenols in the pyrolysis of lignin with and without calcium salt additives. APs: Alkyl-phenols, MPs: Methoxy-phenols.

The compositions of bio-oil from pyrolyzing lignin with and without the additives of CaCl_2_, Ca(OH)_2_, and Ca(HCOO)_2_ were analyzed using GC/MS. A total of five categories (including 46 kinds of compounds) were detected in this experiment, as detailed in Table 1. As seen in Figure 3a, the bio-oils of all lignin samples were mainly composed of phenols, with aldehydes, ketones, ethers, and aromatic hydrocarbons also being present. It is well known that phenolic compounds are the major products from lignin pyrolysis [26,27,28], which are derived from the cleavage of the ether bonds between the lignin-building units and the further cracking or replacing of the side chains of these units [29]. As compared to pyrolysis without a catalyst, adding calcium salts dramatically increased the content of phenols in the bio-oils, from 73.65% to 78.03%, 79.29% and 79.86%, respectively. Therefore, it is obvious that all these additives had a significant catalytic effect on the phenol yield in lignin pyrolysis; this effect was more pronounced with Ca(HCOO)_2_ additives. Moreover, one can see that the additions of Ca(OH)_2_ and Ca(HCOO)_2_ generally decreased the productions of aldehydes and ketones, which were obtained from the C–C cleavage in the alkyl side chains with the –CH_2_OH or –COOH groups [30,31]. Consequently, it is supposed that the catalytic effect of calcium salts could reduce the unsaturated degree of the liquid products from lignin pyrolysis. Besides, the formation of ethers and aromatic hydrocarbons was attributed to the cracking of the ether and methoxy linkages in the alkyl side chains and to the changes in substitution pattern of the aromatic rings [22,29]. Both CaCl_2_ and Ca(HCOO)_2_ increased the production of aromatic hydrocarbons and decreased that of ethers, whereas calcium formate facilitated more of an increase in the production of aromatic hydrocarbons (from 1.96% to 8.73%) and a decrease in the production of ethers (from 16.35% to 7.56%).

Figure 3b shows the variation in contents of major phenols from pyrolyzing lignin with and without calcium salt additives. The major phenols were phenol, alkyl-phenols (e.g., methylphenol, ethylphenol, and xylenol), methoxy-phenols (e.g., methoxy-methylphenol, methoxy-ethylphenol, methoxy-propylphenol, and methoxy-propenylphenol), guaiacol, and vanillin. From Figure 3b and Table 1, it can be seen that calcium salt additives greatly elevated the production of phenol and alkyl-phenols, and the Ca(HCOO)_2_ additive caused the largest increase in the formation of these two chemicals (14.89% for phenol and 21.09% for alkyl-phenols). The result implies that these additives promoted the cracking of lignin into monophenols, which was beneficial to improve the chemical reactivity of bio-oil. Furthermore, one can also see that all the additives in this experiment tended to decrease the productions of guaiacol and vanillin in an order CaCl_2_ < Ca(OH)_2_ < Ca(HCOO)_2_, the yields of which were originally about 26.1% and 1.79%, respectively, in the bio-oils of pure lignin pyrolysis. This indicates that calcium salts facilitated the decomposition reactions of substances with a guaiacyl phenol structure such as guaiacol and vanillin. According to Hosoya (2008) [32], lignin-derived compounds can undergo secondary reactions at high temperatures. Therefore, this led us to the conclusion that adding calcium salts promoted secondary reactions in the pyrolysis process of lignin, which is in line with the theory that guaiacyl phenols tend to decompose to monophenols. Regarding the methoxy-phenols content, the addition of CaCl_2_ and Ca(OH)_2_ slightly increased it by a few percent compared to its production (19.79%) in lignin pyrolysis, while Ca(HCOO)_2_ effectively reduced it to 12.01%. Figure 3 also demonstrates that Ca(HCOO)_2_ was more influential than the same amount of CaCl_2_ or Ca(OH)_2_ on facilitating the production of value-added chemicals (e.g., phenol, alkyl-phenols, and aromatic hydrocarbons) in bio-oils from lignin pyrolysis. On the basis of former researches [14,33], when adding modest amounts of formic acid in the form of a metal salt, deoxygenation, addition, and reduction reactions are promoted during fast pyrolysis of lignin, facilitated by the hydrogen generated from the thermal decomposition of the formate. In addition, this is in good agreement with the results of this study: That catalysis of calcium formate is more conducive to reducing the oxygen content and increasing the saturated degree of the products of lignin pyrolysis.

### 3.3. The Characteristics of Char

**Figure 4:**
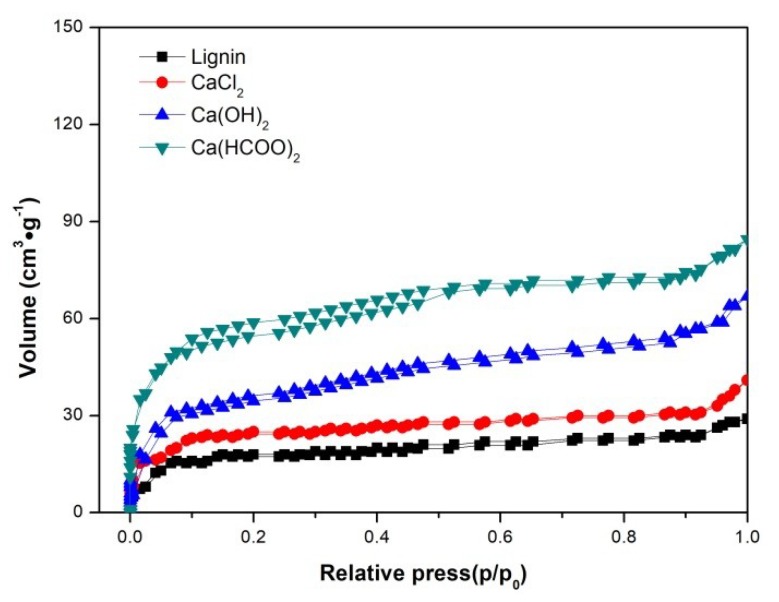
N_2_ adsorption–desorption isotherm of chars from lignin pyrolysis with and without calcium salt additives.

As seen in Figure 4, all the samples exhibited a weak absorption in the entire relative pressure range, which demonstrates that there was a relatively small number of micropores and mesoporous in the pyrolysis chars. The lower values of BET surface area and total pore volume (in Table 2) also illustrates that the chars obtained from lignin pyrolysis with or without additives were almost non-porous materials. This, therefore, well coincides with the previous conclusion that chars derived from lignin pyrolysis need the process of further activation before being used as commercial activated carbons [21]. Furthermore, according to IUPAC (International Union of Pure and Applied Chemistry) classification, the diameter of mesopores was between 2 and 50 nm [34]. Thus, the chars (prepared in this experiment) showed the character of mesoporosity, depending on the value of average pore diameter listed in Table 2.

One can also see that chars prepared by lignin pyrolysis with the addition of Ca(OH)_2_ and Ca(HCOO)_2_ had a greater amount of N_2_ absorption, a higher BET surface area, and total pore volume compared to two other kinds of chars in this study. It was seen that adding Ca(OH)_2_ or Ca(HCOO)_2_ during lignin pyrolysis was conducive to improving the adsorption of the obtained chars. As a kind of hydroxide, Ca(OH)_2_ may play the role of chemical activating agent during the carbonization process of lignin, causing more pores in the chars to be created or enlarged. Additionally, the formate was easily decomposed to volatile substances (such as H_2_ and CO_2_) at high temperatures, which can promote the formation of pore structures and enhance the BET surface area of the char from the lignin pyrolysis.

**Figure 5:**
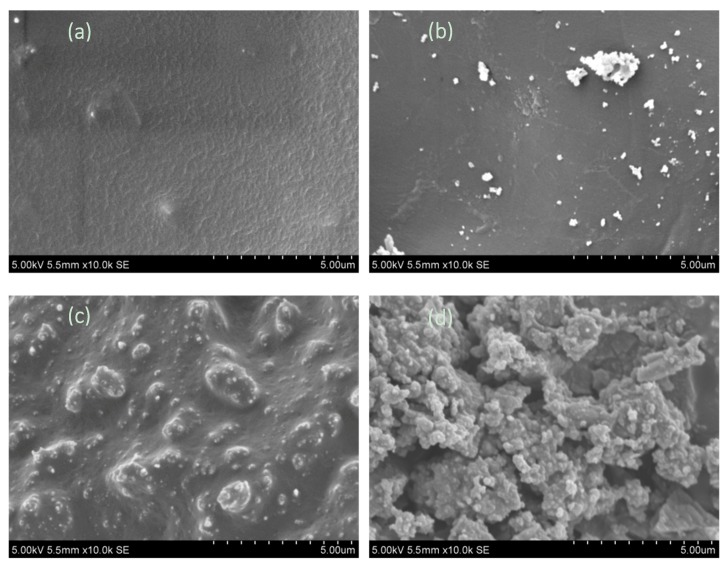
Scanning electron microscopy (SEM) images of chars prepared from (**a**) pure lignin pyrolysis or with the additions of (**b**) CaCl_2_, (**c**) Ca(OH)_2_, and (**d**) Ca(HCOO)_2_.

As shown in Figure 5a, the surface of the char obtained from pure lignin pyrolysis was relatively smooth and had no obvious pores. Hence, that kind of char needed further activation to improve the capacity of absorption. As can be seen in Figure 5b, a large amount of particulate matter was attached to the surface of chars and fewer pores were discovered, which indicates that the added CaCl_2_ still remained after the pyrolysis process and blocked the incipient porosity of the chars. However, the surface was obviously unsmooth in the samples in which Ca(OH)_2_ and Ca(HCOO)_2_ was added, as can be seen in Figure 5c,d. Particularly, more pores were seen in the char produced from the addition of Ca(HCOO)_2_. 

## 4. Conclusions

The catalytic effects of calcium salt additives on the product characteristics of pyrolysis lignin were experimentally investigated and compared. The results show that the catalytic effect of three kinds of calcium salt additives led to the same tendencies in the yields of pyrolysis products, which promoted the bio-oil yields and inhibited the formation of gas and char. Regarding the gas formed through lignin pyrolysis, calcium salt additives increased the concentrations of H_2_ and CH_4_, but decreased the concentrations of CO. The chemicals in bio-oil from pyrolyzing lignin with or without additives mainly consisted of phenolic compounds, while calcium salt additives elevated the production of both phenol and alkyl-phenols and decreased the yields of guaiacol and vanillin in an order of CaCl_2_ < Ca(OH)_2_ < Ca(HCOO)_2_. Furthermore, adding Ca(OH)_2_ or Ca(HCOO)_2_ was more conducive to an increase in the adsorption of chars obtained from lignin pyrolysis. By comparing the catalytic effect of the additives used in this study, it can be seen that adding Ca(HCOO)_2_ in lignin pyrolysis is an effective method to improve the quality of the pyrolysis products and increase the content of value-added chemicals (e.g., phenol, alkyl-phenols, and aromatic hydrocarbons ) in bio-oils.

## Figures and Tables

**Table 1:** The major compositions of bio-oil from lignin pyrolysis with and without calcium salt additives.

No.	Name of Compounds	Relative Content (Area%)			
		Lignin	CaCl_2_	Ca(OH)_2_	Ca(HCOO)_2_
Phenols		73.65	78.03	79.29	79.86
1	Phenol	3.08	4.39	6.25	14.89
2	2-Methylphenol	0.82	1.98	2.19	3.22
3	4-Methylphenol	0.34	1.18	1.81	3.36
4	Guaiacol	26.1	21.63	20.04	17.68
5	2,4-Xylenol	—	0.56	1.09	0.98
6	2,6-Xylenol	—	—	0.71	1.48
7	2-Ethylphenol	0.78	0.97	1.3	1.61
8	4-Ethylphenol	0.21	5.34	5.78	7.43
9	4-Ethyl-2-methylphenol	0.67	1.98	3.22	3.01
10	2-Methoxy-5-methylphenol	5.93	5.05	5.68	4.96
11	2-Methoxy-4-methylphenol	9.27	9.68	7.67	2.13
12	3-Methylcatechol	1.25	2.18	2.04	3.89
13	3-Methoxycatechol	1.02	0.89	0.98	0.52
14	2-Methoxy-4-ethylphenol	3.55	4.87	5.53	3.73
15	2,6-Xylohydroquinone	—	0.61	0.78	0.68
16	4-Hydroxy-3-methylacetophenone	2.37	3.08	2.1	1.79
17	2,6-Dimethoxyphenol	5.48	2.83	2.62	2.01
18	3-Allyl-2-methoxyphenol	—	0.79	1.58	0.95
19	2-Methoxy-4-propylphenol	—	0.67	0.89	0.73
20	Vanillin	1.79	1.05	0.76	0.62
21	2-Methoxy-4-propenylphenol	1.04	0.79	1.73	0.46
22	cis-Isoeugenol	—	2.05	—	0.19
23	Guaiacylacetone	—	1.22	0.49	0.74
24	Apocynin	1.04	2.91	1.43	1.71
25	Homovanillic acid	0.57	1.02	0.47	1.09
26	Salicylaldehyde	—	0.31	1.03	—
27	Isovanillin	8.34	—	1.12	—
**Aldehydes**		**3.82**	**6.** **02**	**3.** **09**	**1.53**
1	Veratraldehyde	3.82	6.02	3.09	1.53
**Ketones**		**4.22**	**4.78**	**2.01**	**2.32**
1	Acetone	1.02	2.76	—	—
2	3-Ethyl-2-hydroxy-2-cyclopenten-1-one	—	2.02	—	—
3	2-Methyl-2-cyclopentenone	1.08	—	2.01	0.92
4	3-Methyl-2-cyclopentenone	2.12	—	—	0.78
5	Methycyclopentenolone	—	—	—	0.62
**Ethers**		**16.35**	**8.61**	**14.19**	**7.56**
1	4-Methoxystyrene	1.62	—	2.08	1.21
2	4-Methylanisole	0.36	—	0.8	0.86
3	Veratrole	9.02	4.22	8.2	2.45
4	4-Ethylveratrol	3.81	2.92	1.08	2.03
5	4-Ethyl-1,2-dimethoxybenzene	—	0.97	1.64	1.01
6	Methylisoeugenol	1.54	0.5	0.39	—
**Aromatic** **Hydrocarbons**		**1.96**	**2.56**	**1.42**	**8.73**
1	Toluene	—	1.21	0.87	2.63
2	Benzene	—	0.18	—	0.32
3	2-Isopropyltoluene	0.41	1.03	—	2.22
4	3-Isopropyltoluene	0.22	—	0.55	2.23
5	Naphthalene	0.81	—	—	0.21
6	Anthracene	0.52	0.14	—	—
7	2,4-Dimethyl styrene	—	—	—	1.12

**Table 2:** The specific surface area and pore structure of chars from lignin pyrolysis with and without calcium salt additives.

Samples	S_BET_	Total Pore Volume	Average Pore Diameter
	(m^2^·g^−1^)	(cm^3^·g^−1^)	(nm)
Lignin	40.62	0.035	3.078
CaCl_2_	62.16	0.051	3.091
Ca(OH)_2_	135.68	0.115	3.012
Ca(HCOO)_2_	175.31	0.152	3.005
